# D2-40 A Helpful Marker in Assessment of Lymphatic Vessel Invasion in Carcinoma of Breast

**DOI:** 10.30699/IJP.2020.114511.2245

**Published:** 2020-12-20

**Authors:** Zeinab Vosough, Shima Golbini, Majid Sharbatdaran, Akramossadat Hosseini

**Affiliations:** 1 *Student Committee Research, Babol University of Medical Sciences, Babol, Iran*; 2 *Cancer Research Center, Health Research Institute, Babol University of Medical Sciences, Babol, Iran*; 3 *Clinical Research Development Center, Shahid Beheshti Hospital, Babol University of Medical Sciences, Babol, Iran*

**Keywords:** Breast, Carcinoma, Monoclonal antibody, D2-40

## Abstract

**Background & Objective:**

Breast cancer is the most common malignancy among Iranian women and worldwide. Lymphatic vessel invasion (LVI) was found to be an independent prognostic factor in many carcinomas, including invasive carcinoma of the breast. The aim of this study was to compare the hematoxylin and eosin (H&E) staining method and use of the immunohistochemical (IHC) marker ofD2-40, for evaluation of LVI in breast carcinoma specimens.

**Methods:**

The study was conducted on 50 patients undergone surgery between the years 2010 and 2015 in Rohani Hospital, Babol, Iran with invasive carcinoma of the breast with Census sampling method. LVI was assessed by two surgical pathologists, using H&E- stained sections and two IHC markers, i.e., D2-40 and CD31.

**Results:**

LVI was detected in 25 (50%) patients by H&E and in 14 (28%) patients by D2-40. Twelve out of 25 patients with positive LVI in H&E were confirmed by D2-40 and 2 out of 25 patients with negative lymphatic vessel in H&E. Only one case showed weak staining of CD31 proving LVI. These results showed a significant difference between the H&E staining and D2-40 IHC study for LVI detection (*P*=0.004).

**Conclusion:**

The D2-40 IHC marker is helpful in the diagnosis and confirmation of LVI in invasive carcinoma of the breast. CD31 is not suitable for the evaluation of lymphatic vessels.

## Introduction

Breast cancer is the most common malignancy in women worldwide, particularly in Iran. Breast cancer is the second leading cause of cancer death in developed countries (15.4%) after lung cancer, and it is the fifth leading cause of cancer death in Iran like any other part of the world. The five-year overall survival rate is estimated to be 72% in women and 60% in men. This survival rate is significantly affected by the stage and number of positive lymph nodes (1).

Lymph node metastasis is the main independent prognostic factor, and the lymphatic system is the major route for tumor dissemination (2). When lymph nodes are free from the tumor, the prognosis is good (10%-20% mortality), and the effect of adjuvant chemotherapy is less prominent than in lymph node-positive cases. Therefore, defining a practical indication for neoadjuvant therapy is an important issue, and a favorable prognostic treatment effect should overcome distress, side effects, and cost of adjuvant therapy (3).

The term lymphovascular invasion is used whenever blood or lymphatic vessels are invaded by tumoral cells (4). In fact, it has been proved that tumoral cells can trigger the growth of tumor-associated lymphatic vessels and eventually enter them (5).

A brief survey through the literature reveals that several studies have been conducted on lymphatic vessel invasion (LVI) of variable tumors. The importance of LVI in breast cancer was first described by Teel in 1964 (6). LVI was found to be an independent prognostic factor in lymph node-negative (3) or -positive (7) breast cancer patients.

In the past, hematoxylin and eosin (H&E) staining was the only method for the evaluation of LVI. It was somehow problematic due to fibrosis and fixation artifact, which resulted in false positivity (8). Therefore, the utilization of immunohistochemistry (IHC) markers drew researchers’ attention. Many studies have suggested that using ancillary techniques increases the detection rate of true LVI (9, 10).

 D2-40 is a monoclonal antibody detecting podoplanin, mucin-type transmembrane glycoproteins on the endothelium of lymphatic vessels (11).

This marker has been used by many studies in variable malignancies (2, 8, 12, 13). CD31 is a protein encoded by PECAM-1 gene and is mainly used for the identification of endothelial cells in many studies (14).

The aim of our study was to compare the H&E method and use of the IHC marker of D2-40, for evaluation of LVI in breast carcinoma specimens.

## Material and Methods

The study was conducted on patients with invasive carcinoma of the breast who had undergone surgery between the years 2010 and 2015 in Rohani Hospital, Babol, Iran, and their formalin-fixed paraffin blocks of the tumor were available. The inclusion criteria were having invasive carcinoma with available paraffin block which included a rim of normal breast tissue in tumor margin. Those cases that diagnosed as carcinoma in situ or presented with extensive tumor necrosis in all slides were excluded from the study. Clinicopathological data, including age, gender, tumor stage, and grade, were retrieved from standard reports, which were prepared according to the American Joint Committee on Cancer (AJCC), 7^th^ edition.

**Immunohistochemistry**

To highlight lymphatic and blood vessels, two 3-4 µm thick sections were prepared from each block (one block per case), and IHC staining for D2-40 and CD31 (Dako, Glostrüp, Denmark) was performed. Sections were dewaxed at 60°C in an oven for about one hour, and then they were put in xylol and rehydrated through a descending concentration of ethanol. For antigen retrieval, sections were microwaved for 15 minutes in ethylenediaminetetraacetic acid (EDTA) buffer (pH=9). Sections were left at room temperature for 15 minutes to cool down. They were washed in tris-buffered saline (TBS) for five minutes and incubated in 3% H_2_O_2_ in dark humid condition. After that, they were washed in TBS for five minutes. Sections were incubated with primary antibody for 60 minutes at room temperature and with secondary antibody for 30 minutes. Sites of binding were detected by a 10-minute incubation with diaminobenzidine (DAB). 

IHC and archived H&E slides were reviewed by two pathologists, not knowing the pathology report. Data were imported in SPSS 20 (SPSS Inc., Chicago, Ill., USA); chi-square, Fisher’s exact, and *t* tests were performed for statistical analysis.

## Results

The current study was conducted on 50 female patients with invasive carcinoma of the breast, including 47 ductal and 3 lobular carcinomas, who had undergone a mastectomy. The mean age was 47.40±12.35 years ranging from 19 to 74. The clinicopathologic characteristics of the tumors are shown in Table 1.

**Table 1:** Clinicopathological characteristics of the cases

Tumor characteristic	Number of cases (percentage %)
Grade1* Grade2 Grade3	7(14) 37(74) 3(6)
Stage1 Stage2 Stage3	7(14) 15(30) 28(56)
LVI present in H&E LVI absent in H&E	25(50) 25(50)
LVI present in D2-40 LVI absent in D2-40	14(28) 36(72)
LVI present in CD31 LVI absent in CD31	1(2) 49(98)

*3 missed data due to invasive lobular carcinoma of the breast

**Fig. 1:**
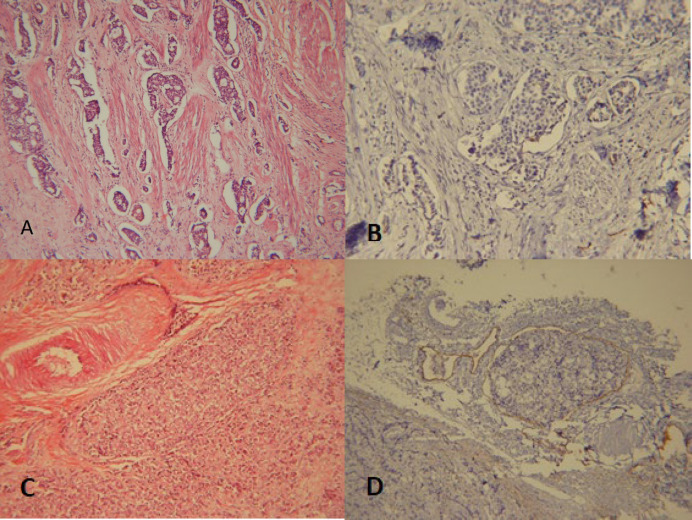
A. Retraction artifact (H&E method x100). B. Retraction artifact proved by D2-40 IHC (x100). C. Tumor emboli filling the lumen (H&E method x100). D. Tumor emboli proved by D2-40 IHC method (x100)

Abbreviations: H&E, hematoxylin and eosin; IHC, immunohistochemistry.

Table 2 shows that 13 cases with the identification of LVI in H&E slides were not confirmed by D2-40, indicating rather mis/over-interpretation (false positive rate: 0.36) of the H&E method mainly caused by retraction artifact (Figures 1A and 1B). 

Two cases in the negative H&E group had LVI in D2-40 stained sections (false negative rate: 0.14). These cases were the result of the presence of tumor emboli that filled the lumen entirely (Figures 1C and 1D)

The H&E method had 0.85 sensitivity and specificity of 0.64. The difference between these two methods was significant (*P*=0.004). 

**Fig. 2:**
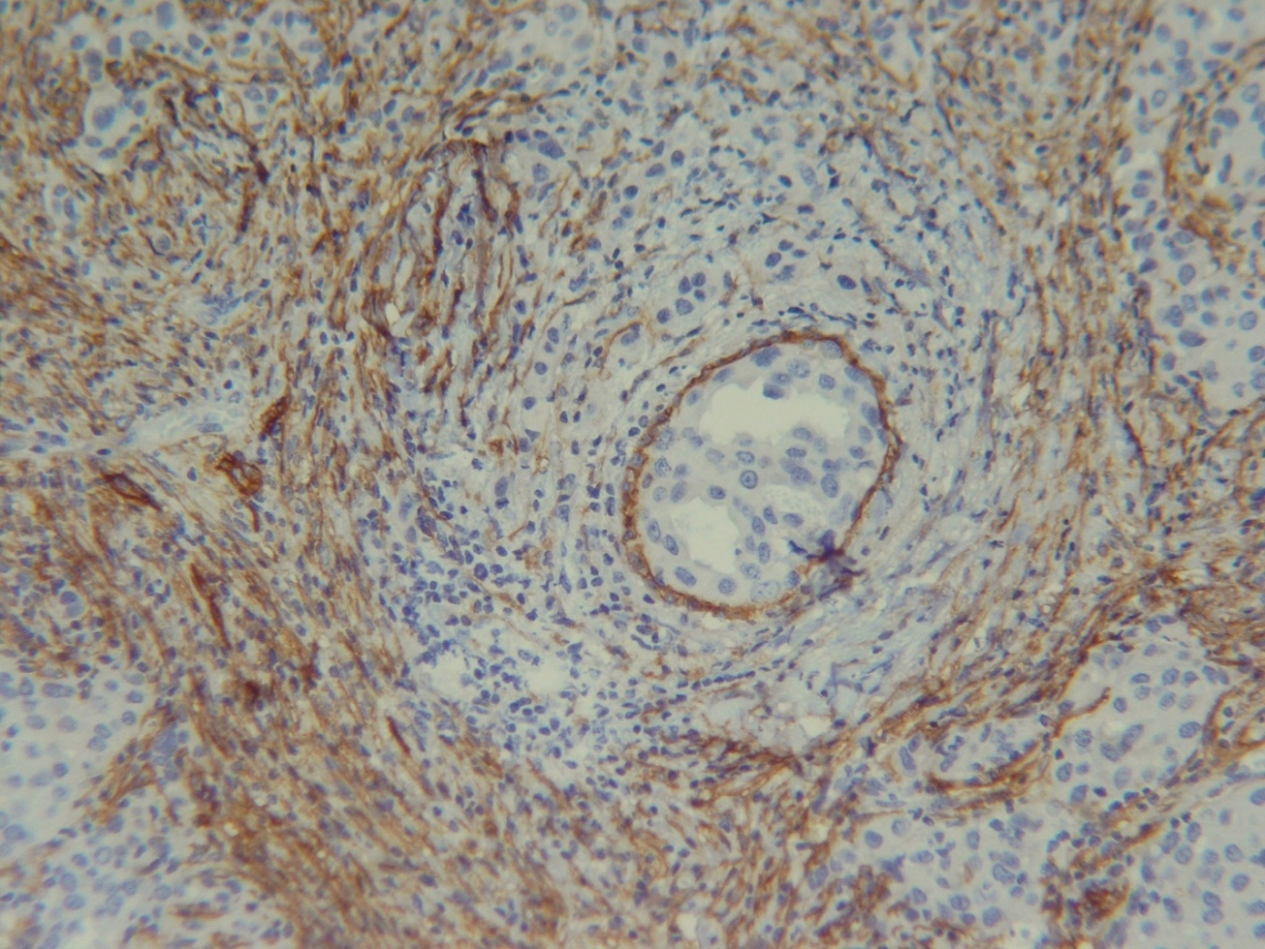
D2-40 IHC in myoepithelium (×100)

Abbreviations: IHC, immunohistochemistry.

The myoepithelial cell layer could be stained non-specifically in D2-40 stained sections, which can cause false positivity in the case of in situ carcinoma (Figure 2). However, we assessed the areas of invasive carcinoma without in situ component to solve this problem.

The assessment of agreement between these two methods was done using Cohen’s kappa, and the calculated kappa value was 0.4, which showed fair agreement.

All the cases, which were negative in D2-40, also showed no staining in the CD31 IHC study. Only one case, which had LVI proved by D2-40, was stained by CD31.

As shown in Table 3, no significant difference was detected between the tumor stage or grade and LVI by D2-40.

.

**Table 2:** Lymphovascular invasion detection by H&E and D2-40 IHC methods

	LVI* present in H&E	LVI absent in H&E	Total
LVI present in D2-40	12	2	14
LVI absent in D2-40	13	23	36
Total	25	25	50

*lymphovascular invasion

Abbreviations: H&E, hematoxylin and eosin; IHC, immunohistochemistry.

**Table 3:** Correlation between clinicopathological characteristics and D2-40 staining results

Clinicopathologic characteristics	D2-40 positive Number of patients (%)	D2-40 negative Number of patients (%)	P-value
Grade 1	2(15.4)	5(14.7)	0.61
Grade 2	10(76.9)	27(79.4)	
Grade 3	1(7.7)	2(5.9)	
Stage 1	1(7.7)	6(16.7)	0.24
Stage 2	4(28.6)	11(30.6)	
Stage 3	9(64.3)	19(52.8)	

## Discussion

Breast cancer is the most common malignancy in women around the world. LVI refers to the invasion of lymphatic or blood vessels in the peritumoral area by tumor emboli (15). LVI has an independent prognostic value, and the routine assessment of LVI is part of the tumor pathology report (16). In our study, we used both H&E and D2-40 IHC methods for the evaluation of LVI. Based on the results, there was a significant difference between these two methods. More than half of the cases, which were interpreted as LVI in H&E-stained sections, were not confirmed by D2-40 immunostaining. This can be explained with retraction artifact resulted from fixation, and also two cases with D2-40 positive LVI were not detected in H&E slides. 

This result could be due to the tumor embolism, which completely filled the lumen of the lymphatic vessel. In addition, with the H&E method, vascular invasion, either lymphatic or blood, could be detected and it can't be decided whether it is a lymph vessel or a blood vessel. For this reason, both CD31 (which stained blood vessel endothelium) and D2-40 were used. There was only one CD31 positive case in the D2-40 positive group and none in the D2-40 negative group. Consequently, the interfering role of blood vessel invasion was absent in this study, and the observed significant difference could be trusted. The results of another study using D2-40 and CD31 are in agreement with our findings. It introduced D2-40 as a useful marker for lymphatic invasion and CD31 for blood vessel invasion (14). 

In the present study, 28% of the cases had LVI detected by D2-40. These results were consistent with those other similar studies. In a study conducted by Gujam *et al.* (17), 35% of the cases had LVI detected by D2-40, and Mohammed Rabab *et al.* (18) reported 47% for the same measure. The former study divided patients according to their clinicopathologic characteristics and assessed LVI by H&E and D2-40 methods in each group. In our study, the tumor pathologic staging and histologic grading were obtained. No significant relationship was identified between the tumor grade or stage and LVI detected by D2-40.

A quick survey through literature revealed that D2-40 has been announced by numerous studies as a helpful marker in LVI detection (17). He *et al.* (19) used this marker for LVI assessment in 255 patients and stated that “the presence of LVI was significantly associated with adverse disease-free survival.” 

In contrast, a study conducted on 124 cases of stage I lung adenocarcinoma showed no prognostic effect for LVI detected by D2-40 staining method. In other words, patients with LVI detected by D2-40 did not have a significantly poorer outcome compared to LVI negative cases (20).

In addition, this marker has been used by many studies performed on other malignant neoplasms, in which the same results were achieved. Lai *et al.* stated that compared to the H&E staining, the utilization of D2-40 for LVI assessment increased the positive rate significantly in colon cancer (8). Another study on the small rectal neuroendocrine tumor showed higher LVI detection in D2-40 IHC compared to H&E (20.6% vs 6.9%) (21). A study on seminomatous testicular cancer suggested that LVI detection can be optimized by D2-40 staining method (12). 

In the present study, the results showed no significant correlation between the cancer stage or tumor grade and LVI detected by D2-40; however, in a study conducted on 303 patients with node-negative invasive breast carcinoma, LVI invasion detected by D2-40 correlated significantly with the tumor size and histologic grade (22). This discordance was possibly explained due to the small sample size, which is the main problem of the current study, or this could be due to the separation of node-negative patients in the above-mentioned study. 

Another error which could be encountered in our study was the positive staining of the myoepithelial cell layer in D2-40 IHC method, which can cause false positivity in cases of in situ carcinoma. However, we assessed the areas of invasive carcinoma without in situ component to solve this problem. In addition, the pattern of staining in lymph vessels is strong continuous membranous, and in the myoepithelial cell layer is weak-to-moderate with granular pattern (23). Rabban JT *et al.* suggested using myoepithelial markers, such as p63 and D2-40 simultaneously, to distinguish tumor emboli from in situ carcinoma (24). In our experience, the growth pattern of tumoral cells surrounded by D2-40 positive cells can help to distinguish carcinoma in situ from LVI. If the suspected area is within the invasive part of the tumor, and definite signs of in situ component are absent, it is probably interpreted as LVI. 

##  Conclusion

In conclusion, the use of the D2-40 IHC marker is helpful in the diagnosis and confirmation of LVI in invasive carcinoma of the breast. CD31 is not helpful in the identification of lymphatic invasion.
